# Next Generation Sequencing in Newborn Screening in the United Kingdom National Health Service

**DOI:** 10.3390/ijns5040040

**Published:** 2019-11-05

**Authors:** Julia C. van Campen, Elizabeth S. A. Sollars, Rebecca C. Thomas, Clare M. Bartlett, Antonio Milano, Matthew D. Parker, Jennifer Dawe, Peter R. Winship, Gerrard Peck, Darren Grafham, Richard J. Kirk, James R. Bonham, Anne C. Goodeve, Ann Dalton

**Affiliations:** 1 Sheffield Diagnostic Genetics Service (SDGS), Sheffield Children’s NHS Foundation Trust, Western Bank, Sheffield S10 2TH, UK; elizabeth.sollars@nhs.net (E.S.A.S.); rebecca.thomas@sch.nhs.uk (R.C.T.); clare_gladding@yahoo.co.uk (C.M.B.); amilano@sidra.org (A.M.); matthew.parker@sheffield.ac.uk (M.D.P.); Jennifer.Dawe@sch.nhs.uk (J.D.); Peter.Winship@sch.nhs.uk (P.R.W.); Gerrard.Peck@lwh.nhs.uk (G.P.); darrengrafham@gmail.com (D.G.); Richard.Kirk@sch.nhs.uk (R.J.K.); a.goodeve@sheffield.ac.uk (A.C.G.); 2 Division of Pharmacy, Diagnostics and Genetics, Sheffield Children’s NHS Foundation Trust, Western Bank, Sheffield S10 2TH, UK; Jim.Bonham@sch.nhs.uk; 3 Department of Infection, Immunity & Cardiovascular Disease, Faculty of Medicine, Dentistry & Health, University of Sheffield Medical School, Beech Hill Road, Sheffield S10 2RX, UK

**Keywords:** newborn screening, next generation sequencing, automation

## Abstract

Next generation DNA sequencing (NGS) has the potential to improve the diagnostic and prognostic utility of newborn screening programmes. This study assesses the feasibility of automating NGS on dried blood spot (DBS) DNA in a United Kingdom National Health Service (UK NHS) laboratory. An NGS panel targeting the entire coding sequence of five genes relevant to disorders currently screened for in newborns in the UK was validated on DBS DNA. An automated process for DNA extraction, NGS and bioinformatics analysis was developed. The process was tested on DBS to determine feasibility, turnaround time and cost. The analytical sensitivity of the assay was 100% and analytical specificity was 99.96%, with a mean 99.5% concordance of variant calls between DBS and venous blood samples in regions with ≥30× coverage (96.8% across all regions; all variant calls were single nucleotide variants (SNVs), with indel performance not assessed). The pipeline enabled processing of up to 1000 samples a week with a turnaround time of four days from receipt of sample to reporting. This study concluded that it is feasible to automate targeted NGS on routine DBS samples in a UK NHS laboratory setting, but it may not currently be cost effective as a first line test.

## 1. Introduction

Newborn screening (NBS) programmes worldwide provide early diagnosis and intervention for infants affected with rare disorders which, if untreated, are associated with progressive deterioration, disability and potentially fatal outcomes [1]. In the UK, NBS is carried out through measurement of biomarkers in a dried blood spot (DBS) taken 5–8 days after birth [2,3,4,5,6,7,8]. Currently, DNA testing is only undertaken as a second line test in the NBS protocol for cystic fibrosis, as part of the follow-up diagnostic protocol for suspected cases of medium-chain acyl-coenzyme A dehydrogenase deficiency (MCADD), isovaleric acidaemia (IVA) and glutaric aciduria type 1 (GA1) and for further characterisation of a subset of suspected haemoglobinopathy cases [3,6,9]. However, the advent of next generation sequencing (NGS) has resulted in attempts to expand the use of DNA sequencing in NBS to improve diagnostic and prognostic utility [10,11,12,13]. Several key features of NGS make it a potentially powerful technology in NBS. A single assay can be used for a range of disorders regardless of whether a biochemical marker is available; simultaneous analysis of large numbers of genetic loci and samples can drive down costs; and laboratory processes can largely be automated. The increasing trend toward provision of genetic laboratory services in larger centralised units in the UK has the potential to enable greater access to high throughput NGS technology. Thus, it is timely and relevant to investigate the potential of NGS in NBS in a UK NHS context.

### 1.1. Next Generation Sequencing in Newborn Screening: Genome Wide or Targeted Assays?

Routine NGS in healthy newborns has predominantly gained attention through studies proposing whole exome sequencing (WES) or whole genome sequencing (WGS) as a first line screening test [12,13]. These studies have shown that WES and WGS from DBS samples are technically feasible; that WGS can identify a wider range of disorders than current NBS methods and may, in some cases, yield fewer false positives and that, although ethical concerns exist, there is considerable interest in it from parents of healthy newborns in the postpartum period [12,13,14].

However, using a targeted NGS panel, where only a subset of genetic loci is analysed rather than a WGS approach, has advantages over WGS. Compared to WGS/WES, targeted approaches minimize the amount of sequence to be covered, reducing data processing time and data storage cost, thus reducing the cost per sample and turnaround time. The inclusion of loci relevant to additional screening disorders could likely be achieved rapidly through NGS panel expansion where necessary.

Two possible targeted approaches can be defined: the entire coding sequence of a selected set of genes can be analysed or analysis can be limited to a panel of known pathogenic variants. The key trade-off in defining how targeted to make any NGS-based NBS assay is that of ease of interpretation versus diagnostic sensitivity. Targeted NGS, especially if only a panel of known variants is analysed, has the benefit of limiting the number of variants of uncertain significance found and the time required for interpretation. However, clinical sensitivity may be unacceptably reduced by only targeting a known panel of variants. Given these considerations, this study took a semi-targeted approach, with the entire coding sequence of a selected set of genes analysed.

### 1.2. Applications of NGS in NBS as an Adjunct Test or Primary Screening Test

Next generation sequencing has the potential to benefit NBS in two modalities, namely, a) as an adjunct test to enhance the utility of current NBS protocols or b) as a primary (first line) screening test used to detect disorders deemed suitable for NBS, but with no existing biochemical marker. The approach described in this study could be used in either modality.

As an adjunct test, DNA sequencing can aid clarification of ambiguous or borderline biochemical screening results. Adjunct genetic testing may also improve the prognostic utility of NBS results, particularly for inborn errors of metabolism, in which variable presentation is a key feature [15,16,17,18,19,20,21,22,23,24,25,26]. Currently, the correct treatment option can be difficult to determine in asymptomatic screen-positive infants [16,22,23]. This can lead to less effective treatment or to unnecessary medicalisation of healthy infants. Adjunct DNA sequencing can pinpoint those screen positive cases which have a mild variant of the disorder and may not require any clinical intervention, such as IVA cases with the benign c.941C>T variant or MCADD cases with the mild c.199T>C variant [25,26]. It also enables precision medicine, for example, in selecting optimal treatment for cystic fibrosis patients [27,28,29].

Alternatively, DNA sequencing may be used as a first line NBS test, particularly for disorders not amenable to biochemical analysis. A timely rare disease diagnosis can prevent the cost and distress associated with an extensive diagnostic odyssey [30,31,32,33]. In addition to the impact on the health and wellbeing of the affected newborn, many rare diseases are heritable, and rapid diagnosis of an infant proband can thus have an impact on relatives’ reproductive decisions [33]. The number of candidate NBS disorders may increase in the future as therapeutic progress opens up new avenues for treatment of rare disorders currently deemed untreatable [34,35,36,37,38].

### 1.3. Development of a Rapid Targeted NGS Assay Utilising DBS DNA

As the collection and analysis of the DBS is at the core of the UK NBS programme, any novel NBS assay must work robustly on this sample [2,3,4,5,6]. Liquid capillary heel prick sampling alternatives are available and would potentially yield better quality DNA than DBS. However, their introduction would require a major change to the UK NBS process, which is not currently anticipated. Targeted NGS from DBS samples has been previously described, but no targeted automated assay is currently available at scale, at a cost, and in a sufficiently fast turnaround time to be appropriate for NBS in a UK National Health Service context [10,11,39,40,41]. Previous work has described manual DNA extraction and library preparation from DBS, automated DNA extraction from DBS followed by manual NGS library preparation or protocols only suitable for the processing of relatively small numbers of samples at once [11,41]. However, DNA in DBS is stable and analytical validity is independent of the baby’s age, condition, feeding or gestation. As such, DBS samples are highly suitable for NBS using targeted NGS. The current study evaluated the technical feasibility and cost of automated, high throughput, fast turnaround, targeted NGS on DBS samples in the UK using a targeted AmpliSeq NGS panel assay and Ion Torrent (semiconductor) sequencing [42].

## 2. Materials and Methods 

### 2.1. Sample Collection

The DBS samples were taken by an experienced midwife from fingertips of adult healthy volunteers who were not affected by any of the disorders analysed and did not have a family history of these disorders. All subjects gave their informed consent for the sharing of anonymised data outside the study. The study was conducted in accordance with the Declaration of Helsinki, and the protocol was approved by the London-Brent Research Ethics Committee (REC reference 14/LO/1715) and the UK Health Research Authority (IRAS ID 159179). The DBS samples were stored at room temperature for up to two weeks prior to extraction and sequencing. Six-millimetre disks were punched from Guthrie cards (Perkin Elmer 226) using the Panthera DBS Puncher (Perkin Elmer) into 0.8 mL 96 well plates (Thermo Fisher Scientific, Altrincham, UK). The DNA contamination of the punch head through repeated punching of DBS disks was found not to be an issue (Figure S1).

### 2.2. DNA Extraction

For extraction of DNA from venous blood, Qiagen DSP DNA kits were used with the Qiasymphony liquid handling platform (Qiagen, Manchester, UK). For extraction of DNA from DBS, the United States Centers for Disease Control and Prevention (CDC) DBS DNA extraction method [43] was modified and automated using a custom program on the Biomek FXp liquid handling platform (Beckmann Coulter, Brea, CA, USA) (Figure S2). Single 6 mm DBS punches in a 0.8 mL 96 well plate covered with a pierceable adhesive plastic seal were used as the input material. Following the addition of the final 60 uL of Qiagen Elution Solution, plates were covered with a non-pierceable adhesive plastic seal and incubated at 99 °C for 15 min. The eluate was then transferred into a new 96 well plate using the Biomek FXp liquid handling platform.

### 2.3. Panel Design and Validation

A custom targeted Ion AmpliSeq panel (WG_IAD48658, hereafter referred to as the “NBS2” panel) was designed to cover all coding regions of the following genes associated with disorders screened for in the UK: *ACADM* (Medium Chain Acyl Co-A Dehydrogenase Deficiency), *PAH* (phenylketonuria), *TSHR* (congenital hypothyroidism), *CFTR* (cystic fibrosis) and *HBB* (sickle cell disease). The panel also covers 3′ untranslated regions (UTRs) of these genes and extends 5 bp either side of each exon. The total size of the panel was 50.054 kb (see Supplementary Materials Data S2, NBS2 panel. BED file for genomic coordinates).

Validation of the panel was carried out according to the American College of Medical Genetics (ACMG) Guidelines for Targeted Sequencing [44], with library preparation carried out manually according to the standard Ion AmpliSeq protocol (Thermo Fisher Scientific; [45]). Sequencing of two high-confidence cell line DNA truth sets was undertaken on the Ion S5 sequencer (Thermo Fisher Scientific), namely, “Genome in a Bottle” (GIAB, sample NA12878, Corriell), with truth set of variants generated by Zook et al. 2014 [46], and the Human Reference Genome (HuRef; sample GM25430, Corriell), with truth set of variants generated by Mu et al. 2015 [47]. Thirty-two DBS samples from healthy controls were also sequenced on the Ion S5 sequencer. In addition, 66 paired venous blood and DBS samples from 33 healthy controls were sequenced on the Ion PGM sequencer (Thermo Fischer Scientific, Altrincham, UK) to assess concordance of variant calling (defined as the percentage of variants matching) between DBS and venous blood DNA samples.

### 2.4. Semi-Automated Library Preparation

Semi-automated library preparation was carried out using the AmpliSeq library preparation protocol (Thermo Fischer Scientific) modified for automation using the Biomek FXp and Biomek NXp liquid handling platforms (Beckmann Coulter) (Figure S3). Three microlitres of undiluted DBS DNA extracted using the automated CDC DBS DNA extraction method was used immediately after extraction in the initial AmpliSeq reaction, and 2 uL HiFi Mastermix and 5 uL primer pool were added to this in 96 well plates using the Biomek Fxp liquid handling platform. The “NBS2” AmpliSeq panel used is made up of two primer pools; as such, two reactions were set-up per sample. After amplification (two minutes at 99 °C, followed by 22 cycles of 15 s at 99 °C and four minutes at 60 °C), the two pools were combined manually and 2 μL FuPa was added manually. Samples were incubated at 50 °C for 10 min, 55 °C for 10 min, and 60 °C for 20 min. Within an hour of the end of this incubation, 4 μL Switch solution, 2 μL DNA Ligase and 1.5 μL of a unique molecular barcode were added to each sample using the Biomek NXp automated liquid handling platform. This reaction was incubated at 22 °C for 30 min, followed by heat inactivation at 72 °C for 10 min. At this stage, library quality and molarity were assessed (TapeStation 2200, Agilent). Library products with sizes between 150 bp and 450 bp were included in molarity determination. Equimolar library pooling was carried out using a custom spreadsheet application and the Biomek NXp liquid handling platform. Assessment of the concentration of input DBS DNA samples or the molarity of each initial AmpliSeq library pool (after the first amplification step) were not found to improve performance during routine high throughput processing; therefore, these steps were omitted from the final process.

### 2.5. Chip Loading and Sequencing

Chip loading was carried out using the Ion Chef liquid handling platform (Thermo Fisher Scientific), with 96 NBS2 panel libraries loaded per Ion 540 chip. Seven high throughput sequencing runs were carried out: four on the Ion S5 sequencer and three on the Ion S5 Prime sequencer (Thermo Fisher Scientific). The run plan had the following parameters: analysis parameters: default; reference library: hg19; target regions: NBS2 panel BED file; hotspot regions: none; read length: 200 bp; flows: 500; base calibration mode: default. The plugins used were coverageAnalysis, DataExport and variantCaller.

### 2.6. Data Analysis

Read mapping was performed automatically in TorrentSuite (v5.2 for S5 runs and v5.8 for S5 Prime runs). Indel re-alignment was carried out using the command-line version of TMAP (ThermoFisher) using v5.2 for S5 runs and v5.4 for S5 Prime runs. Variant calling was then performed using the command-line version of TVC (ThermoFisher) using v5.2 for S5 runs and v5.4 for S5 Prime runs, and v.5.8 for the DBS versus VB comparison. The following parameters were changed from default to improve variant calling sensitivity: Minimum variant score (quality) = 10, minimum allele frequency = 0.1, minimum coverage = 10, min coverage each strand = 0, maximum strand bias = 1 and strand bias *p*-value = 0, homopolymer maximum length = 9 bp. Variant calling was performed on the designed panel regions (WG_IAD48658), decomposed and normalised using vt (v0.5772), and then restricted down to the NBS2 panel regions (exon ± 5 bp) using the Bedtools (v2.25.0) “intersect” command [48,49]. Base-by-base coverage across the NBS2 panel was ascertained using the Sambamba (v0.6.7) “depth base” command [50].

Sequencing quality of the NBS2 panel regions was evaluated using “% bases with 50x coverage” cut-offs of 90% (samples below this were deemed to have failed), 97% (samples in this range may have gaps) and 99.5% (samples above this are unlikely to have substantial gaps). Comparison of variant calls from cell lines to their respective truth sets was performed using the Bcftools (v1.3.1) “isec” command [50,51].

## 3. Results

### 3.1. Validation of the Custom Targeted Ion AmpliSeq Panel for Use on DBS DNA

The genes targeted by our custom NGS panel were selected for proof of principle following discussion with the National Screening Committee and alignment with the existing UK screening programmes; development of alternative screening programmes by NGS was not a project aim. The designed panel covered 3′ UTRs of these genes and extended 5 bp either side of each exon. No additional intronic regions were targeted to maximise coverage of exonic regions and to facilitate rapid and unambiguous interpretation in the context of NBS. We used the Ion Torrent S5 XL platform, as the rapid amplicon-based library preparation enables a fast turnaround time; it has the potential for good cost effectiveness and it affords a high degree of sample multiplexing and, thus, throughput.

The NBS2 panel was validated using cell line truth sets as well as DNA from both venous blood (VB) and DBS (Figure S4 and S5). The overall analytical sensitivity of the panel across both cell line truth sets (GIAB and HuRef) was 100%, specificity was 99.96% and Matthew’s correlation coefficient was 0.963, with 85 unique variants matching the truth sets sequenced. There were 130 correctly called true positive variants, 49,905 true negative reference calls and no false negative (missed) calls. Out of a total of ten false positive calls across the GIAB and HuRef samples, eight were in the same intronic short tandem repeat (STR) region upstream of exon 8 of the *TSHR* gene, which would be excluded in a targeted analysis in which only exons ± 5 bp would be analysed. All true variants in these cell line samples were SNVs; no data on analytical sensitivity or specificity for indels are available. Low coverage areas were limited in number. There was one small exonic region that had an average coverage below 30× (part of *TSHR* exon 10) and another that had an average coverage between 30 and 50× (*CFTR* exon 1), with all other regions having an average coverage of ≥50× (Figure S4).

**Figure 1:**
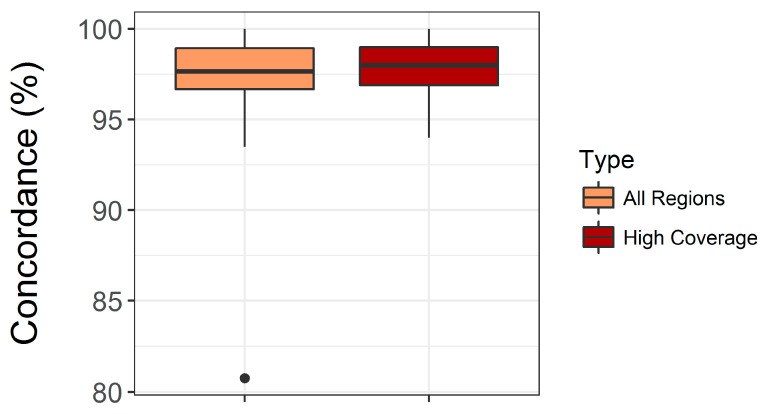
Concordance of variant calls, dried blood spot (DBS) versus venous blood. Variant calls were compared in both the whole panel (“All Regions”) or in regions with coverage over 50× only (“High Coverage”). *n* = 33.

Mean concordance of variant calling between sequencing from VB DNA and DBS DNA was 96.8% (99.5% in regions of >30× read depth, based on 3166 variant calls in 33 paired samples), with a total of 215 unique variants displaying concordance between venous blood and DBS samples (Figure 1; Table S1). Discordant variants were not correctly called for a variety of reasons including low allele frequency causing false positives and STR regions causing misalignments. Out of a total of 3166 variant calls, four false positive SNVs were called in DBS (of which three were in areas with a coverage ≤30×), two false positive SNVs were called in VB (both in areas with a coverage ≤30×), 29 SNVs were missed in DBS (DBS false negatives; of which 26 in areas with a coverage ≤30×), and 38 SNVs were missed in VB (VB false negatives; of which 36 in areas with a coverage ≤30×). There were five false positive indel calls in DBS (one of which was in an area with a coverage ≤30×; the other four calls were all at the same position in a poly-T tract) and no false positive indel calls in VB. There was one indel present in two individuals for whom paired DBS and VB samples were analysed (Phe508del, the common pathogenic variant in the *CFTR* gene). This indel was correctly called from both VB and DBS samples for both individuals. The panel met the ACGS acceptance criteria for NGS panels (Supplementary Materials Section S4, Figure S4, and Table S1; [44]) and was validated as suitable for sequencing from DBS DNA as well as from VB DNA.

### 3.2. Performance of Semi-Automated Next Generation Sequencing from DBS

Libraries were prepared from healthy control venous blood (VB) samples manually and using a semi-automated process. This semi-automated process was found to result in improved uniformity and an improved percentage of reads on target compared to manual library preparation (Figure S6).

**Figure 2:**
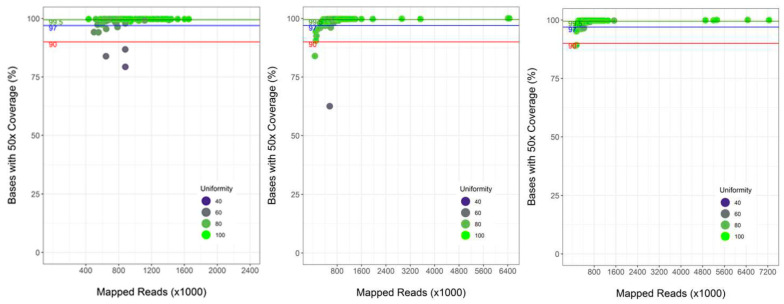
Quality of three high throughput sequencing runs (96 samples per run) from dried blood spots (Ion S5 Prime). Libraries were prepared using the semi-automated AmpliSeq library preparation process.

Libraries were also prepared from DBS using semi-automated processes for DNA extraction and library preparation. Four runs of 96 samples were carried out on the Ion S5 sequencer (Figure S7) and three runs of 96 samples were carried out on the Ion S5 Prime sequencer (Figure 2). Automated DNA extraction yielded DNA of a satisfactory concentration (0.79–1.77 ng/µL) in 96/96 samples in run 1 and 95/96 samples in run 2 (S5 runs). Across the three S5 Prime runs, 78% of samples (225/288) had 50× coverage for ≥99.5% of bases, 92% of samples (264/288) had 50× coverage for ≥97% of bases and 98% of samples (281/288) had 50× coverage for ≥90% of bases.

### 3.3. Timeline for High Throughput Sequencing from DBS

**Figure 3:**
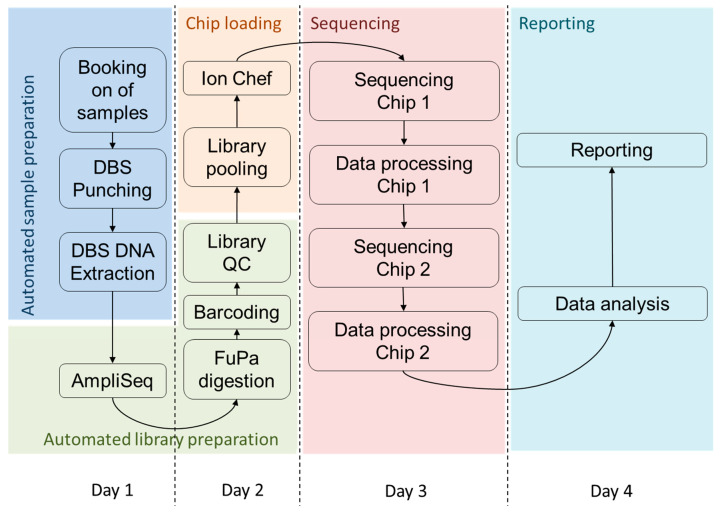
Timeline for automated fast turnaround time NGS from DBS. Booking on of samples and DNA extraction were carried out on Day 1. Automated AmpliSeq library preparation was initiated on Day 1 and PCR was run overnight. FuPa digestion (manual pooling and FuPa enzyme addition), automated barcoding, automated library QC, and automated library pooling were carried out on Day 2. The Ion Chef was used for automated loading of libraries onto sequencing chips overnight on Day 2. Sequencing and data processing were carried out on Day 3, with data analysis (assuming all samples were run in parallel) and reporting on Day 4.

Using this semi-automated sequencing pipeline, up to 192 (2 × 96) libraries can be prepared from DBS DNA in one day, with the entire process from booking on of samples to reporting of results fitting into a four-day turnaround time (Figure 3; Figure S8). Booking on of Guthrie cards, DBS punching, DNA extraction and the initial step of AmpliSeq library preparation were carried out on day 1; FuPa digestion, barcode ligation, library quality control, equimolar library pooling and chip loading on day 2; sequencing and data processing on day 3; and data analysis and reporting on day 4. Repeated daily in the context of a seven-day service, this pipeline was capable of processing over 1000 samples per week. The cost of this semi-automated process including punching of samples, DNA extraction, library preparation and sequencing was calculated at approximately £62.41/sample (including labour) or £60.58/sample (excluding labour), based on a full run of 96 samples per sequencer chip (with a repeat rate of 3/96 samples).

## 4. Discussion

We set out to explore the feasibility of targeted NGS from DBS as a high throughput NBS assay using a semi-automated protocol. The only step of NGS library preparation that was not automated was the addition of the FuPa enzyme, which is highly viscous and, in our hands, not amenable to automated liquid handling. A fully automated library preparation process would be preferable for assay robustness and avoidance of operator error, although the difference in time required would be negligible. Alternative rapid, potentially fully automatable amplicon-based library preparation methods are now also available for other NGS platforms [52]. Our assay was developed in a UK NHS diagnostic genetics laboratory, and this study provides proof of principle that targeted NGS can be carried out in this setting on a scale and in a timeframe compatible with the demands of routine NBS. However, the cost of the assay is not currently comparable to that of existing biochemical assays used in first line screening.

### 4.1. Assay Performance

The assay has an analytical sensitivity of 100% and analytical specificity of 99.96% (figures are for SNVs only; the number of indels called was too low to estimate figures for this variant class). When comparing VB and DBS sequencing, we found good concordance between sample types (96.8% in all areas or 99.5% excluding areas with ≤30× coverage). The vast majority of the discrepancies between VB and DBS samples were false negative SNV calls (in both DBS and VB) due to the low coverage; there were generally similar numbers of erroneous variant calls from DBS and VB; however, a larger number of false positive indels were called in DBS (five) than in VB blood samples (none). Therefore, when considering implementation of an Ion Torrent NGS assay in NBS, it may be appropriate to either (a) analyse only a known set of variant loci and exclude indels or (b) confirm every indel found before reporting. The Ion Torrent platform is known to have issues with calling indels [53]. We have not tested the assay on samples with copy number variants (CNVs) or structural variants (SVs). As such, our method has the potential to provide a high analytical sensitivity and specificity when targeting SNVs, but its effectiveness for indels, CNVs and SVs is unknown. Detection of CNVs or SVs would require careful panel design, and hybridisation capture-based NGS library preparation methods may be more appropriate [54,55,56,57,58].

Finally, we did not include intronic regions in the assay, despite the presence in these regions of known pathogenic variants with a relatively high prevalence for selected disorders including cystic fibrosis (CF) and phenylketonuria (PKU) [59,60]. This approach was taken to minimise the size of the NGS panel and reduce the detection of variants of unknown significance (VUSs). Similarly, repetitive sequences including the poly-TG poly-T tract of the *CFTR* gene were not included, as such regions are likely to give poor results using our assay and have limited utility in predicting severe disease in newborns. The 3′UTRs of the genes targeted were included in our panel, but as interpretation of variants in UTRs is difficult it may be preferable to exclude them. The effects of this approach on clinical sensitivity remain to be evaluated.

### 4.2. Turnaround Time of the Assay

A robust fast turnaround time is essential for any NBS assay. In total across the three runs, we estimated that 92% (with ≥97% of bases having 50× coverage) to 98% (with ≥90% of bases having 50× coverage) of samples had sequencing data of sufficient quality for clinical interpretation within a four-day turnaround time, without the need for repeat testing or Sanger sequencing confirmation of variants found. Any samples requiring repeat testing would have a turnaround time of at least eight days.

For adjunct genetic testing, the NGS assay described here could be carried out within a turnaround time compatible with current UK requirements [61]. However, a faster turnaround time would be required if the assay were to be used as a first line test. Currently, in the UK, the target time of the first clinical appointment for screen positive babies is by 17 days of age (metabolic disorders), with longer times to first clinical appointment acceptable for CF (28 days of age) and for screen negative results (issued within six weeks) [61]. This is particularly challenging in the light of the fact that the DBS sample is not taken until 5–8 days of age. If the DBS sample were taken at birth, this would allow for earlier initiation of the laboratory assay, enabling current target times to the first appointment to be met.

This study provides a proof of principle that a targeted NGS assay on DBS samples can be automated such that over 1000 samples a week can be processed. This throughput would require a seven-day lab service, with two 96 sample runs set-up daily and overlapping four-day periods of sample processing. A doubling of the capacity to 2000 samples per week is feasible with duplicate key equipment (Ion Chef and S5 Prime sequencer). In the UK, the average newborn screening laboratory tests around 50,000 babies tested per year, an achievable throughput using our setup [61].

### 4.3. Cost of High throughput NGS for NBS

The cost of this assay was approximately £71.14/sample. This included booking on samples, DBS punching, DNA extraction, the NGS assay, equipment and labour. Variant interpretation and reporting costs are excluded as an automated process is likely required (not developed in this study). Our cost estimate was based on a full run of 96 samples per sequencer chip, required for economies of scale. Given the current cost per sample (approximately £25 per baby including equipment, staffing, biochemical assays and reporting, Jim Bonham, personal communication), the NGS assay developed here is unlikely to be deemed cost effective as a first line NBS test in the UK in its current form.

However, sampling at birth could result in a major cost saving compared to current practice. This would avoid the community midwife visit at day 5–8, the costliest element of UK NBS, in all hospital births (97.7% of births, 2013 [62]). Secondly, NGS may offer a cost saving by the avoidance of carrier status reporting in recessive disorders. In NBS cases in which only a single pathogenic variant is found in a gene with an autosomal recessive inheritance pattern, this variant could be filtered out before raw data are reported on by the analyst. In cystic fibrosis screening in a UK context, where the laboratory guide to NBS for cystic fibrosis states: “The UK protocol is intended to minimise detection of unaffected heterozygotes”, such filtering may be considered desirable [5]. This could lead to a reduced requirement for second bloodspots at day 21 (~84% of which currently result in a “cystic fibrosis not suspected” report [61]) and a reduction in requests for genetic counselling in unaffected carriers. This would improve the cost effectiveness of the screening pathway. In other screening contexts or populations with high consanguinity, reporting carrier status may be considered beneficial, and a reporting pipeline appropriate to the screening context should be designed.

### 4.4. Expansion of Newborn Screening through Targeted NGS as a First Line Test

As the cost of targeted NGS falls, it has the potential to allow for expansion of NBS to disorders for which a suitable screening technology is currently not available. Targeted sequencing rather than a genome- or exome-wide approach enables addition of screening programmes to continue on a disorder-by-disorder basis, as is currently preferred by the UK National Screening Committee, rather than in response to any technological imperative [63,64,65,66,67]. A targeted analysis also reduces the risk of incidental findings [68,69,70,71]. Targeted rather than genome scale sequencing may also help prevent any drop in screening uptake due to the fact of parental fears of inappropriate use of genetic data [72,73].

The degree to which any NBS-based NGS assay should be targeted requires careful thought. Analysing the entire coding sequence of selected genes has the advantage of increasing sensitivity, as all coding variants are likely to be detected, including rare or previously unseen variants. However, variant interpretation is time consuming and the return of VUSs is undesirable. Reporting of VUSs carries a risk of unnecessary medicalisation of false positive or ambiguous screening cases, the avoidance of which is key to UK screening policy [7,61]. This is especially relevant for disorders with a high degree of genetic or allelic variation or with variable penetrance [74,75,76]. Variant interpretation in NBS would be complicated by the absence of phenotype information or family segregation studies. It may be possible to ameliorate these issues by developing adjunct biochemical or RNA testing on either DBS or liquid blood samples and by developing a thorough understanding of genotype–phenotype correlations in candidate NBS disorders [77,78,79,80,81,82,83,84].

An alternative way of using NGS in NBS is to target specific genetic variants, rather than analysing the entire coding sequence of genes [11,81,82]. A major advantage of this approach is that the number of newborns in whom a VUS is detected can be reduced to zero. However, this approach may limit clinical sensitivity, as rare or previously unseen variants would go undetected. As new treatment options for rare diseases become available, studies of genotype–treatment response correlations may inform which subset of pathogenic/likely pathogenic variants are considered actionable and, therefore, which variants to include in any targeted panel [27,85,86,87,88].

The analytical platform studied here could be used for the analysis of either the entire coding sequence of a set of genes, or of only a selected set of variants. Further studies on the two approaches would be needed to assess the optimal balance of clinical sensitivity and ease of interpretation.

## 5. Summary

Here, we have demonstrated that it is technically feasible to perform targeted NGS on DBS DNA with a sufficiently high throughput and fast turnaround time that the assay could be used in a UK NBS pathway. To assess clinical sensitivity and specificity, the implementation of any targeted NGS assay for NBS should be carefully evaluated through pilot studies in the population to be screened [82,83]. This assessment was not an aim of our study. Further work would be required using samples from both affected patients (with as wide a range of variant types as is relevant to the disorder to be screened for) and a cohort representative of the population to be screened. Although the aim of this study was to assess the potential of targeted NGS in routine NBS, targeted NGS from DBS may also be applicable in a range of other clinical pathways [89,90,91]. This assay thus provides a basis for further work to develop cost effective, fast, high throughput targeted NGS from DBS.

## Supplementary Materials

The following are available online at https://www.mdpi.com/2409-515X/5/4/40/s1, Figure S1: Absence of DNA contamination on punch head; Figure S2: Automated DNA extraction protocol for dried blood spots; Figure S3: Semi-automated protocol for AmpliSeq library preparation; Figure S4: Average coverage for 30 dried blood spot samples across exons in the NBS2 panel; Figure S5: Percentage of amplicons with 100× coverage, reads on target and uniformity in dried blood spot (DBS) versus venous blood (VB) samples; Figure S6: Sequencing quality metrics, manual versus automated library preparation; Figure S7: Quality of high throughput sequencing from dried blood spots on Ion S5 sequencer; Figure S8: Detailed timings for automated dried blood spot DNA extraction, AmpliSeq library preparation, Ion Chef chip loading and sequencing; Table S1: Comparison of variant calls from venous blood (VB) and blood spot (DBS) extraction methods for 33 samples, Supplementary Materials 2, NBS2 panel .BED file.
