# Supplementary material for: Next Generation Sequencing in Newborn Screening in the United Kingdom National Health Service
Source: Int J Neonatal Screen. 2019 Nov 5;5(4):40. doi: 10.3390/ijns5040040 (PMC6914376; doi:10.3390/ijns5040040)
Supplement: Supplementary file 1 [file IJNS-05-00040-s001.zip › Supplementary.pdf]

Draft Supplementary data

S1. Absence of DNA contamination on punch head

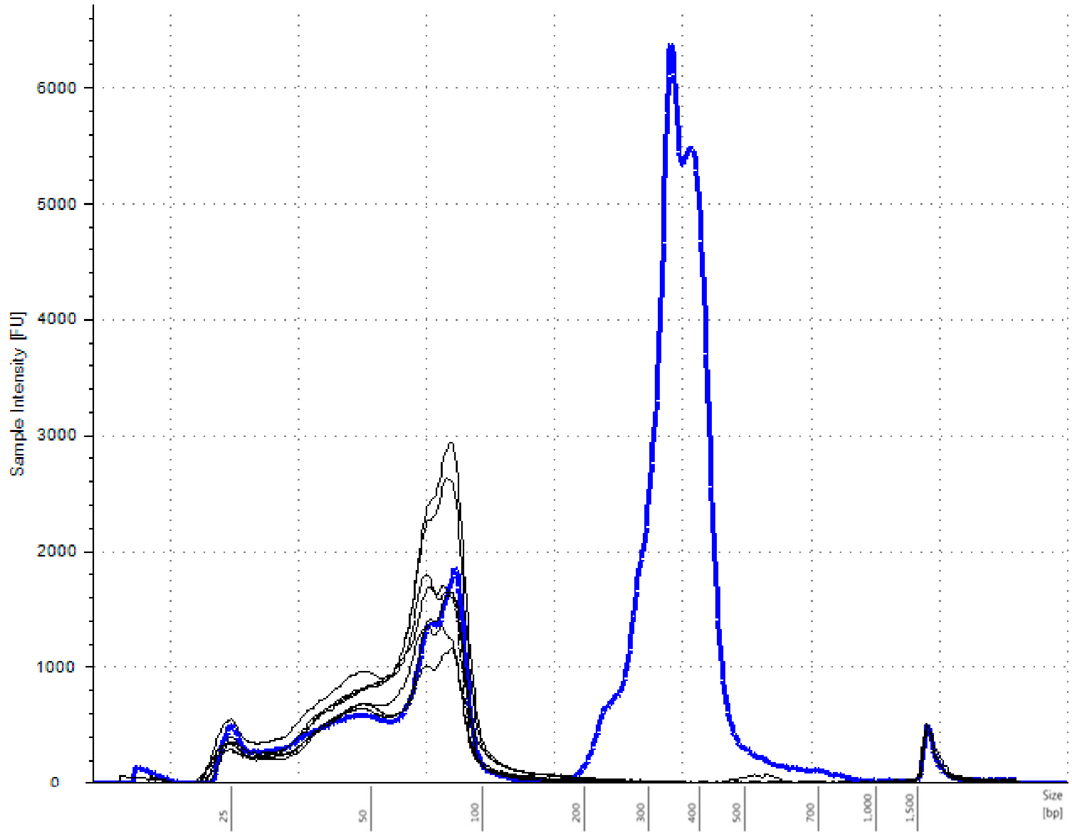

Supplementary Figure 1. Absence of DNA contamination on punch head. TapeStation traces of final libraries prepared from eluate from six standard blank Guthrie card punches (Punch 1 to Punch 6; black lines) vs. a library prepared from eluate from one 6mm dried blood spot punch (blue line).

Blank Guthrie card disks were punched using a punch head that had not been cleaned after a routine day of newborn screening service punching, and subjected to the high throughput DNA extraction and library preparation protocols (as described in Materials and Methods; see main text). When these libraries were assessed on the TapeStation alongside a library prepared from a dried blood spot sample in the same way, a large library peak was detected in the library prepared from a dried blood spot but not in the libraries prepared from blank Guthrie cards. This indicates a negligible amount of amplifiable DNA is present on the punch head. Cross-contamination arising from this is thus considered unlikely.

S2. Automated DNA extraction protocol for Dried Blood Spots

**1. Purification wash x2**		**2. Elution wash**		**3. Elution**
Add 125µl Purification solution		Add 125µl Elution solution		Add 60µl Elution solution
Pipette mix		Pipette mix		Incubate 99°C 15 min
Add 125µl Purification solution		Add 125µl Elution solution		Pulse spin
Pipette mix		Pipette mix		Shake 1400rpm 1 min
Shake 900rpm 100s		Shake 900rpm 100s		Pulse spin
Incubate room temp 250s	x2	Incubate room temp 250s	x2	Transfer 60µl supernatant to plate
Pipette mix<br>Shake 900rpm 100s		Pipette mix<br>Shake 900rpm 100s		
Remove 250µl supernatant	x2	Remove 250µl supernatant	x2	
Wash tips		Wash tips		

Supplementary Figure 2. Automated protocol used on the Biomek FXp for high throughput DNA extraction from Dried Blood Spots.

S3. Semi-automated protocol for AmpliSeq library preparation

**1. AmpliSeq reaction setup (repeat protocol for second primer pool)**	**2. Combine amplified library pools**	**3. Adapter ligation reaction setup**	**4. Equimolar pooling (volumes based on library molarity)**
Transfer 5uL primer pool into reaction plate	Transfer 10uL from reaction plate containing pool 2 into reaction plate containing pool 1	Transfer 210uL Switch solution into tube for mastermix	Transfer appropriate volume of water into pooling tube
Add 2uL HiFi Mastermix	Pipette mix	Transfer 105uL Ligase into tube for mastermix	Transfer appropriate volume of each library into pooling tube
Add 3µl DBS DNA		Pipette mix	Pipette mix
Pipette mix		Add 6uL mastermix (Switch solution plus ligase) to reaction plate	
		Transfer 1.5uL molecular barcode from 96-well plate to reaction plate	

Supplementary Figure 3. Semi-automated protocol used on the Biomek NXp for high throughput AmpliSeq library preparation.

S4. NGS panel validation

Acceptance Criteria

We adhered to the ACGS Guidelines for Targeted Sequencing [44], which state that at least 60 unique variants must be sequenced with no more than a 5% error rate, and that the validation should not contain only commercial cell lines (clinical samples must also be part of the panel validation).

Validation against commercial cell lines

Truth Set 1: Genome in a Bottle
The NBS2 panel shows 100% sensitivity in the GIAB sample, with 74 matching variants. Five false positives were identified; 4 of these lie within an STR (short tandem repeat) region chr14 81574701-81574711 and the final false positive located at chr7: 117250747 was a low-quality variant (QUAL 57) with a coverage of 10. This region would be flagged by the NGS pipeline as a gap.

Truth Set 2: HuRef
The NBS2 panel shows 100% sensitivity in the HuRef sample, with 56 matching variants, no false negatives and five false positives. Four of these were again found within the same STR region as in the GIAB sample. The one remaining false positive was a SNV located at chr20: 55932607 (NB: this variant was also called as a false positive in the NBS1 HuRef sample).

In total, 85 unique variants matched the truth sets in the NBS2 panel. The overall sensitivity across both cell lines is 100%, specificity is 99.96% and Matthew's Correlation Coefficient (MCC) is 0.963.

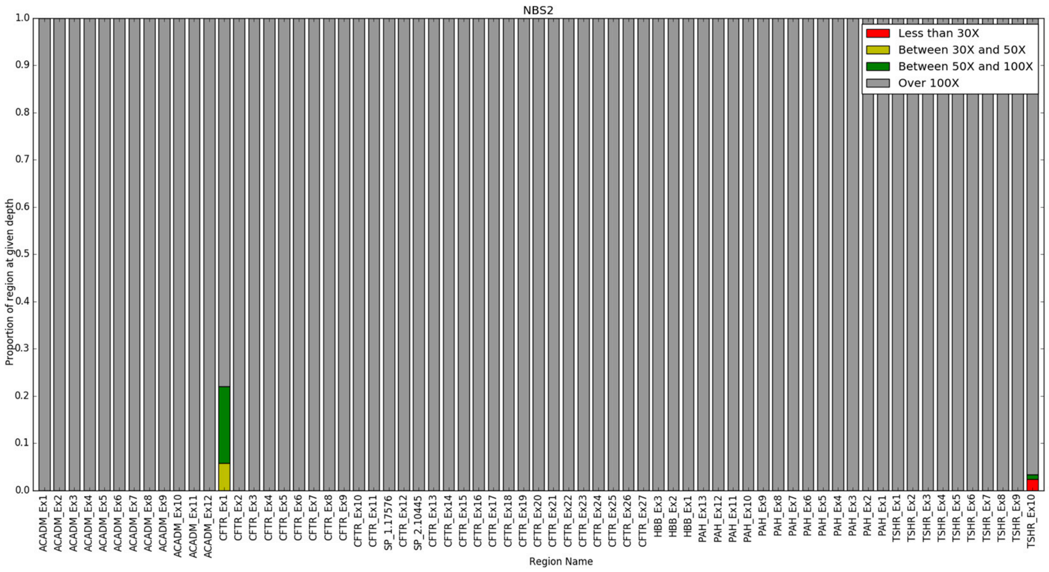

Supplementary Figure 4. Average coverage for 30 DBS samples across exons in the NBS2 panel. Bars depict percentage of region at various average coverage levels (red= <30X, yellow=30-50X, green=50-100X, grey= >100X)

Concordance of variant calling, DBS vs. VB samples

Sample	No. matching variants	No. unique in DBS	No. unique in VB	No. unique due to low coverage	Concordance excluding low coverage (%)
**BO**	91	0	1		98.9
**BR**	92	1	2	2	98.9
**BS**	102	1	1	1	99.0
**BT**	96	0	0		100.0
**BU**	94	1	2		96.9
**BV**	92	0	0		100.0
**BW**	108	0	1		99.1
**BX**	86	3	3	2	95.6
**BA**	101	0	1		99.0
**BB**	84	20	0	20	100.0
**BC**	98	1	2		97.0
**BD**	113	0	0		100.0
**BE**	101	1	0		99.0
**MB**	93	3	2		94.9
**MA**	97	2	0		98.0
**MC**	99	2	1		97.1
**MD**	83	2	0		97.6
**ME**	106	1	1		98.1
**MF**	103	0	0		100.0
**MG**	88	0	2		97.8
**MH**	67	0	41	40	98.5
**MI**	96	3	0		97.0
**MJ**	85	2	3	2	96.6
**AF**	84	0	2		97.7
**AG**	94	3	0		96.9
**AH**	92	1	3		95.8
**AJ**	79	3	2		94.0
**AL**	98	0	0		100.0
**AM**	93	1	2		95.9
**AN**	90	0	1		98.9
**AO**	98	1	1		98.0
**AP**	101	2	2		96.2
**AQ**	95	2	2		96.0

Supplementary Table 1. Comparison of variant calls from Venous blood (VB) and Dried Blood Spot (DBS) extraction methods for 33 samples.

Many of the discordances had a quality score below 30, and therefore are very likely to be false positive calls. Many of the discordances were located outside of exons. The genotypes of three positions over 13 samples were confirmed using Sanger. Sample 'BX' had an extra DBS variant called at chr7: 117232481 (*CFTR* exon 14) which was not present in the equivalent VB sample; this was confirmed as a true heterozygous variant through Sanger sequencing. The likely cause of this discordance is amplification of only the reference allele in the VB sample. The remaining two positions were confirmed as false positive calls in all samples they were

sequenced in; chr14:81528568 in sample 'MB' having a low QUAL score in the variant call (23), and chr14:81610296 across several samples appearing to have strand bias. Clarification of a further two discrepant variants through Sanger sequencing was unsuccessful as they were located in poly-T tracts.

S5. Quality of Sequencing from Dried Blood Spots vs. Venous Blood

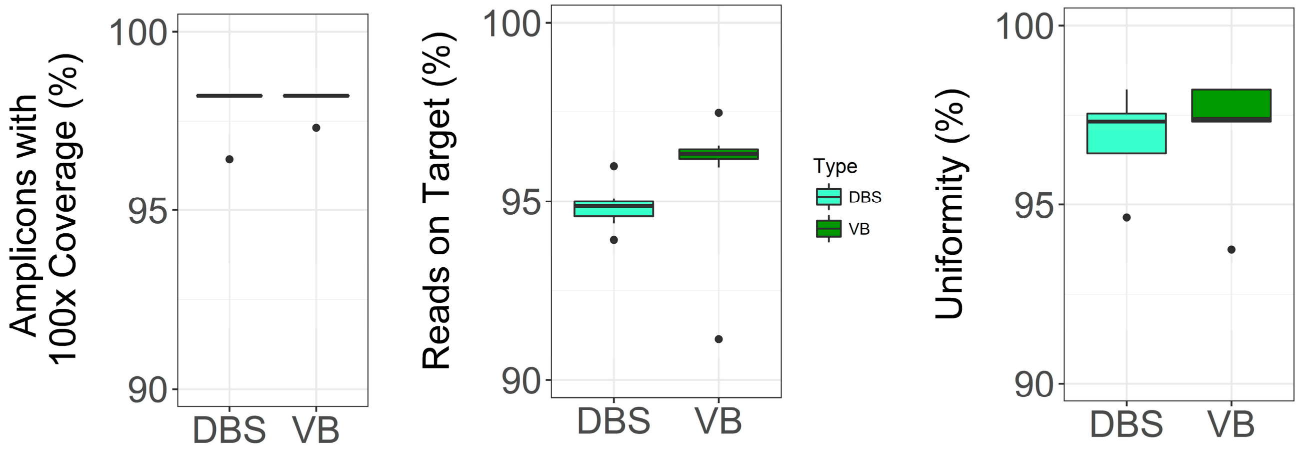

Supplementary Figure 5. Percentage of amplicons with 100x coverage, reads on target and uniformity in dried blood spot (DBS) vs. venous blood (VB) samples

S6. Manual vs. automated sequencing

Sequencing quality metrics indicated that uniformity of reads across amplicons was higher in libraries prepared using automation compared to manually prepared libraries (p=0.0001068*, Wilcoxon Rank Test), as was the percentage of reads on target ($p < 2.2 \times 10^{-16}$, Wilcoxon Rank Test). Libraries prepared using automation were slightly smaller in molarity than those prepared manually but this was not statistically significant and had no impact on sequencing as all libraries are diluted to an approximately identical molarity during final library pooling.

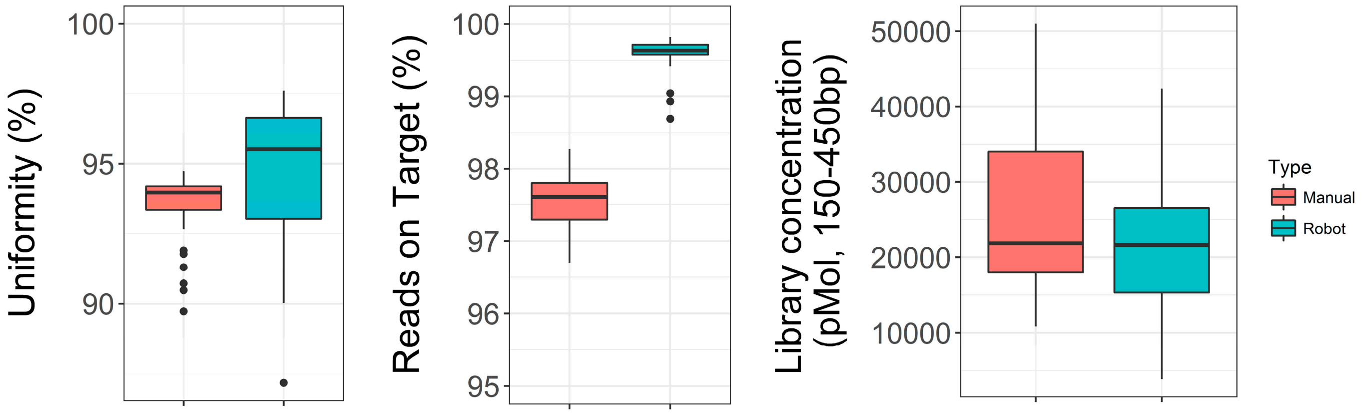

Supplementary Figure 6. Sequencing quality metrics, manual vs. automated library preparation. Sample input was DNA isolated from venous blood.

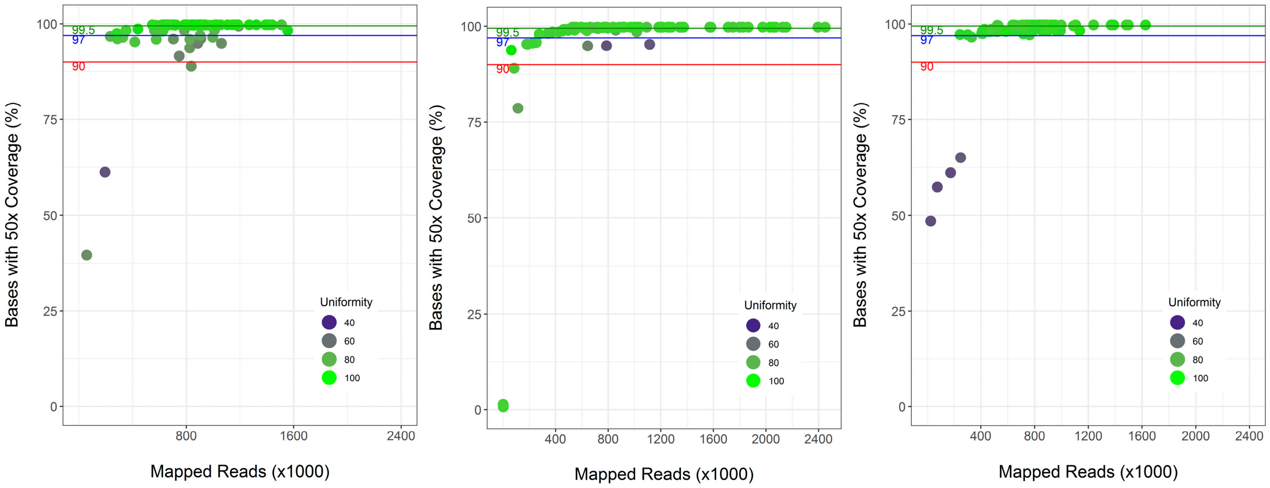

Supplementary Figure 7. Quality of three high throughput sequencing runs from Dried Blood Spots on Ion S5 sequencer. Libraries were prepared using the semi-automated AmpliSeq library preparation process. 96 samples were sequenced on each run.

S7. Timings of high throughput process for two 96-well plates

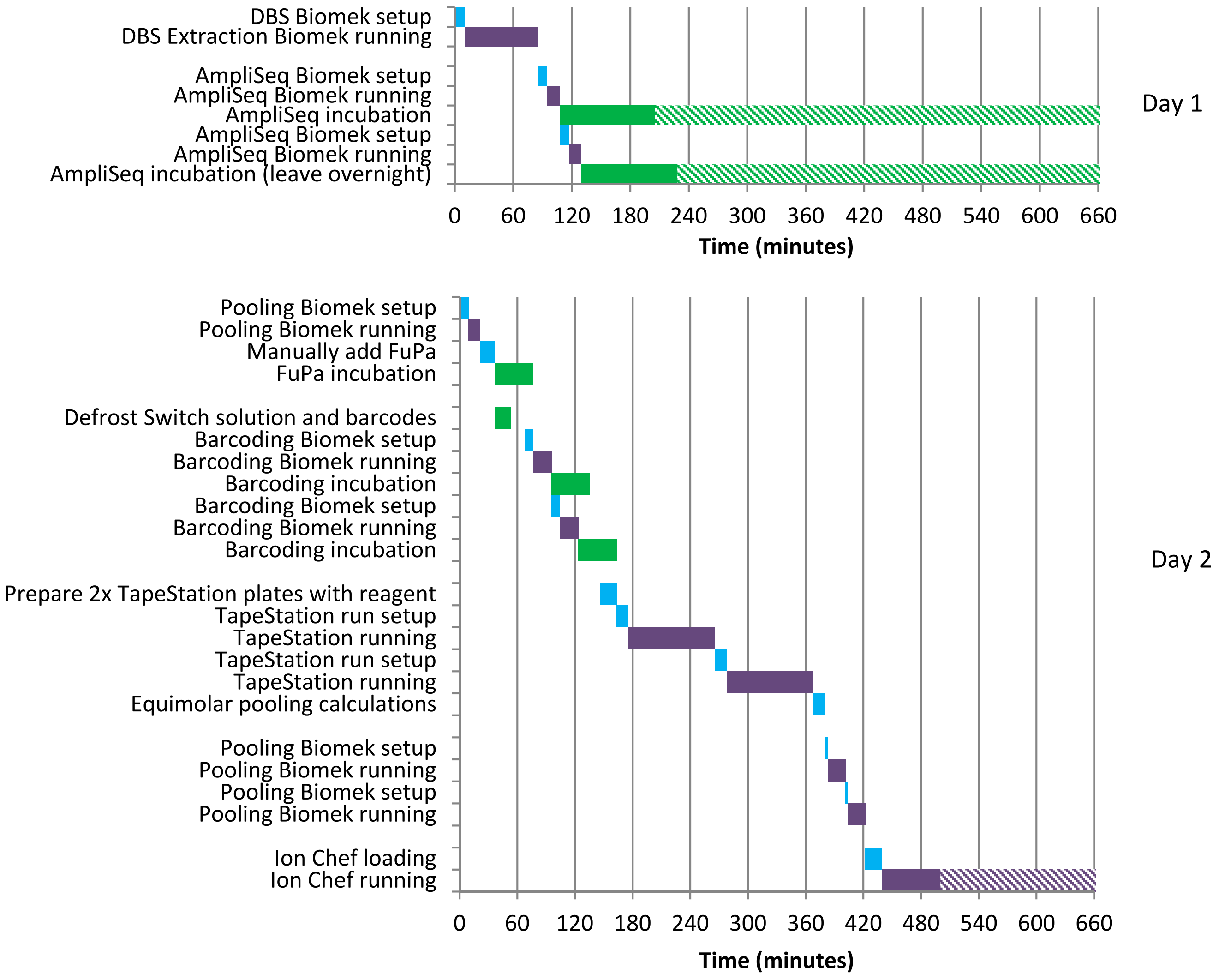

Supplementary Figure 8. Detailed timings for automated DBS DNA extraction, AmpliSeq library preparation, Ion Chef chip loading and sequencing. Purple bars: liquid handling platform running. Blue: manual step. Green: incubation. Dashed bars represent overnight steps.
